# From Cultivation to Application: Unlocking Microalgae’s Potential in Sustainable Food Systems

**DOI:** 10.3390/foods15061108

**Published:** 2026-03-23

**Authors:** Xinyue Guo, Minato Wakisaka, Jiangyu Zhu

**Affiliations:** 1 School of Food Science and Engineering, Yangzhou University, Yangzhou 225127, China; mz120242221@stu.yzu.edu.cn; 2 Food Study Centre, Fukuoka Women’s University, 1-1-1 Kasumigaoka, Fukuoka 813-8529, Japan; wakisaka@fwu.ac.jp

## 1. Introduction

Microalgae, as functional food ingredients with high nutritional value and sustainability, have garnered significant attention for their content of proteins, polyunsaturated fatty acids, vitamins, and antioxidant components [1,2]. Driven by the growing global demand for healthy and sustainable foods, their market potential is substantial. However, their large-scale application still faces numerous challenges. The World Health Organization has repeatedly emphasized the importance of developing sustainable protein sources [3]. As an emerging food, microalgae encounter issues such as high cultivation costs, sensory characteristics not easily accepted by consumers, complex biomass processing technologies, and insufficient long-term storage stability [4,5,6], which severely restrict their industrialization progress and market penetration. Furthermore, large-scale cultivation involves challenges related to water and energy consumption, high medium costs, as well as potential contamination and quality control issues in the production chain, posing ongoing technological and economic hurdles for the industry.

Academia and industry are increasingly focusing on interdisciplinary strategies to address these challenges, aiming to achieve the high-value utilization of microalgae resources. These strategies include utilizing industrial and agricultural waste as low-cost culture media to achieve a circular economy [7], employing mixed or co-culture technologies to enhance biomass yield and product diversity [8], and applying genetic editing and metabolic engineering to directionally improve algal strain traits [9]. In particular, processing microalgal components using methods such as microencapsulation, fermentation, or compounding with other food matrices can improve their flavor, mask undesirable colors, and enhance their functionality and stability within complex food systems. This provides a promising pathway for developing the next generation of nutritionally fortified and environmentally friendly food ingredients.

## 2. Research Topic Overview

Cvetković et al. (Contribution 1) studied the effects of adding blue spirulina powder on the nutritional composition, antioxidant activity, color, texture, and sensory characteristics of sour cherry juice and tomato juice. This research was conducted because spirulina (*Arthrospira platensis*), although rich in protein and antioxidant components, often faces limitations in beverage applications due to consumer acceptance of its odor and taste. The study adopted a “reverse screening” strategy, first screening sour cherry juice and tomato juice as the most acceptable bases from four common juices (apple, sour cherry, tomato, celery) through sensory acceptance tests, followed by systematic analysis. After adding blue spirulina powder (0.8% and 1.6% *w*/*w*), the protein, fat, and energy values of both juices significantly increased, and antioxidant activity was markedly enhanced. Color analysis showed a shift in juice color from red to blue tones, which was especially more pronounced in sour cherry juice. Texture analysis indicated that the hardness and consistency of tomato juice decreased, while the viscosity index increased. Sensory evaluation found that the characteristic fruit or vegetable flavor of the juices weakened after spirulina powder addition, with algal odor and flavor becoming stronger, intensifying with increased addition levels. Despite sensory challenges, the study concluded that existing filling and packaging equipment is still suitable for such products and pointed out that the method of sensory-first substrate screening helps develop functional beverages with greater market potential. In summary, spirulina-fortified sour cherry juice and tomato juice have significant potential for nutritional and functional enhancement, but optimizing their sensory characteristics remains a key direction for future product development.

Amario et al. (Contribution 2) systematically reviewed the current research status of microalgal lipids in the food industry by analyzing 255 publications from 2019 to 2024. The study found that the number of related publications has declined in recent years, with China being the main contributing country. The vast majority of studies were at laboratory scale, focusing on lipid production using green algae through optimization of cultivation conditions. Lipid extraction and characterization primarily employed the Bligh & Dyer/Folch method, gravimetric methods, and GC-MS technology. Bibliometric results confirmed that research on polyunsaturated fatty acids (such as Omega-3 and Omega-6) is most abundant, consistent with their known health benefits; simultaneously, saturated fatty acids also occupy a significant proportion, indicating their potential in food ingredient development. However, the analysis pointed out that, apart from preliminary application evidence in the field of aquatic feed, research on the application of microalgal oil as a substitute for saturated animal fat in the broader food industry remains sparse. In conclusion, microalgal lipids, especially their saturated fatty acid components, have broad prospects in developing sustainable plant-based foods, but current research remains predominantly at the laboratory stage. Future efforts urgently need to expand towards practical application and industrialization research.

Milia et al. (Contribution 3) explored the potential of using abandoned mine water to replace traditional resources for cultivating spirulina (*A. platensis*). This research aimed to address the challenges of high costs and high energy consumption in the industrial production of spirulina by seeking a more environmentally and economically viable cultivation solution utilizing mine water with a constant temperature and ample supply. Experiments were conducted in photobioreactors, comparing the effects of distilled water supplemented with Zarrouk medium (DWZ) and mine water supplemented with the same medium (MWZ) on spirulina growth and biochemical composition. The data showed that spirulina cultivated using MWZ had significantly higher protein content (52.64 ± 2.51 g/100 g dry weight), while the DWZ group accumulated more lipids and carbohydrates. Although mine water alone could not support spirulina growth, after nutrient supplementation, its final biomass and pigment production showed no significant difference compared to the control group. Analysis suggested that the higher sulfate content in mine water might promote protein synthesis. This study demonstrates that using nutrient-rich mine water for spirulina cultivation can not only yield biomass with superior nutritional composition, but also significantly reduce costs associated with water treatment and temperature control, providing a new pathway with practical application prospects for sustainable spirulina production.

Fanari et al. (Contribution 4) focused on the effects of different microalgal species on the physicochemical and sensory stability of high-protein vegetable creams, filling a gap in the study of long-term storage stability of microalgae-fortified vegetable creams. The study selected *Spirulina*, *Tetraselmis chui*, and four strains of *Chlorella vulgaris*, evaluating their impact on the quality of vegetable creams over an eight-month storage period. A multidimensional analytical approach was employed: first, frozen vegetables were used as the base raw material, combined with single-cell components of six microalgae to prepare vegetable creams, ensuring all microalgal formulations met the EU “high protein” standard; after processing steps including cooking, homogenization, and sterilization, samples were stored at room temperature; physicochemical parameters were analyzed instrumentally, microbial indicators were detected via plate count method, and sensory attributes were assessed by a trained panel, both initially and after 8 months of storage. The results showed that microalgal addition enhanced the homogeneity and emulsification stability of the vegetable creams, with all microalgal formulations meeting the “high protein” requirement. Sensorially, they generally enhanced umami and salty perception, with yellow *C. vulgaris* having the least impact on flavor, while *T. chui* significantly altered sensory characteristics due to its strong fishy and shellfish-like flavor. After 8 months of storage, microbial loads in the samples were below safety thresholds, with no detrimental changes in texture, taste, or odor, and shelf life was not negatively affected, but slight browning was observed. Network analysis clearly identified spirulina and some *Chlorella* strains as natural flavor enhancers, while *T. chui* is more suitable for developing novel food concepts.

Zhu et al. (Contribution 5) aimed to valorize fruit waste by exploring the effects of different pretreatment methods on the growth of *Euglena gracilis* and the synthesis of the high-value product β-1,3-glucan to develop low-cost, sustainable culture media for *E. gracilis*. The study selected three types of fruit waste—bagasse, banana peel, and watermelon peel—comparing the effectiveness of three pretreatments, water extraction (WE), high-temperature and high-pressure treatment (HTP), and dilute sulfuric acid treatment (DSA), using commercial HUT medium as a control. The research method was concise and efficient: extracts were prepared from the three fruit wastes using different pretreatments, adjusted for pH and supplemented with a nitrogen source to serve as culture media; after inoculating with *E. gracilis* and culturing for 14 days, cell density, reducing sugar content, cell morphology, photosynthetic pigments, photosynthetic efficiency, and β-1,3-glucan yield were measured, with data verified by statistical analysis. The results showed that DSA pretreatment was the most effective: the cell density of *E. gracilis* in the bagasse-DSA group reached 2.08 times that of the control group, while the banana peel-DSA and watermelon peel-DSA groups reached 1.35 and 1.70 times, respectively; the content of photosynthetic pigments (chlorophyll a, carotenoids, etc.) and photosynthetic efficiency parameters (Fv/Fm, Fv/F0) were significantly enhanced; and β-1,3-glucan yield increased by 23.99%, 12.92%, and 23.38% in the three waste-DSA groups, respectively. The order of effectiveness for the three pretreatments was DSA > HTP > WE, with bagasse showing the most significant response to DSA treatment. The study confirms that fruit waste pretreated with DSA can be used for low-cost and efficient cultivation of *E. gracilis*, achieving a win–win situation for waste valorization and high-value product production, aligning with the concept of a circular economy.

Esteves et al. (Contribution 6) provided a review focusing on co-culture systems of marine oleaginous microalgae such as *Nannochloropsis* spp. with microorganisms, systematically summarizing their interaction mechanisms, biotechnological applications, and potential in the food field, offering a reference for the large-scale application in this area. The study reviewed the relevant literature from 2001 to 2024, focusing on analyzing co-culture models of *Nannochloropsis* with other microalgae, bacteria, and fungi. Interaction mechanisms include metabolite exchange and chemical signaling. The advantages of co-culture are reflected in increased biomass and lipid yields, simplified biomass harvesting, and enhanced wastewater treatment efficiency. Application scenarios cover biofuel production, wastewater purification, and aquatic feed, with particular potential highlighted in the food field: *Nannochloropsis* contains up to 30% dry weight protein with a balanced amino acid profile, is rich in eicosapentaenoic acid (EPA), and is listed in the EU’s novel food catalog, making it a potential alternative protein source and suitable for integration into cellular agriculture to optimize the nutrition of cultured meat. Current challenges include species competition, contamination control, and strain compatibility issues in scaled-up cultivation, necessitating the optimization of culture conditions and screening of non-pathogenic symbiotic microorganisms. Future efforts should focus on exploring optimization of food-grade co-culture systems, improving the digestibility of *Nannochloropsis*, and promoting its application in functional foods and cellular agriculture to contribute to sustainable biotechnology development.

Spínola et al. (Contribution 7) provided a comprehensive review focusing on the chemical composition, diverse biological activities, and multi-sector applications of spirulina (*Limnospira platensis*), offering a thorough reference for its large-scale utilization and in-depth development. By searching the relevant literature up to 2024 in databases such as Google Scholar and PubMed, the study systematically summarized the nutritional composition, bioactive mechanisms, and practical application scenarios of spirulina, while analyzing current research gaps and future development directions. Core content reveals that spirulina is nutritionally rich and biologically active: chemically, it contains 50–70% dry weight of high-quality protein, lipids, polysaccharides, pigments like phycocyanin, as well as various vitamins and minerals; in terms of bioactivity, compounds such as phycocyanin, polysaccharides, and carotenoids possess antioxidant, anti-inflammatory, immunomodulatory, antiviral, anticancer, hypoglycemic, and lipid-regulating effects, which can assist in preventing and ameliorating chronic diseases. Application scenarios are broad: in the food sector, it can serve as a nutritional supplement or natural colorant to enhance product nutritional value and appeal; in the feed sector, it can replace traditional protein sources to improve the growth performance and immunity of livestock, poultry, and aquatic species; in the pharmaceutical sector, it holds potential for development as an adjuvant therapy for chronic diseases, antiviral agents, etc. Current challenges include high cultivation costs, the need to improve the bioavailability of nutrients through pretreatment, and insufficient clinical validation. Future efforts should focus on optimizing cultivation techniques, improving pretreatment methods, conducting large-scale clinical trials, and further expanding its applications in functional foods, novel feeds, and drug development to help address global nutrition and health challenges.

Chen et al. (Contribution 8) studied the effects of conjugating microalgal phenolics and protein on the structure, bioactivity, and bioaccessibility of the conjugates. This research was conducted because *Chlorella vulgaris*, although rich in protein and phenolic components, often faces limitations in functional food and nutraceutical applications due to the low stability and bioaccessibility of these compounds. The study adopted an “extraction-conjugation” strategy, first extracting free phenolics and protein from *Chlorella vulgaris*, followed by the synthesis of conjugates at different ratios (2.5–10%) and systematic analysis. After covalently conjugating the phenolics and protein, the antioxidant activity (including ABTS and ACE inhibitory activities) and bioaccessibility of the conjugates significantly increased. Spectral analysis showed a decrease in the fluorescence intensity and UV–Vis absorbance of the protein, confirming the alteration of the chromophore area. Structural analysis indicated that covalent bonds were successfully formed through Schiff base and Michael addition reactions. In vitro evaluation found that the 2.5% conjugate exhibited the highest bioaccessibility, reaching 2.5 times that of free phenolics. Despite the instability challenges of the original compounds, the study concluded that covalent conjugation is an effective pathway to enhance the bioactivity and bioaccessibility of microalgae-derived compounds and pointed out that elucidating the structure–activity relationship of the conjugates helps develop functional products with greater market potential. In summary, microalgal phenolic–protein conjugates have significant potential for nutritional and functional enhancement, but utilizing these findings to promote the development of high-value microalgae products remains a key direction for future product development.

## 3. Conclusions

Microalgae, as high-nutrient, sustainable functional food ingredients, demonstrate significant potential in enhancing the functionality of foods, such as protein content and antioxidant activity. However, their industrialization still faces multiple challenges, including high costs, low sensory acceptance, and insufficient processing stability. Strategies such as cultivation using waste resources, compounding with food matrices, and optimization of processing technologies can, to some extent, improve their economic viability and applicability; nonetheless, optimizing their sensory characteristics and ensuring their long-term stability remain key to achieving market transformation.

Future research should focus on sensory improvement technologies and enhancing consumer acceptance, developing low-cost and sustainable cultivation and processing systems, and strengthening the functional retention and stability of microalgal components within complex food matrices. Simultaneously, it is necessary to promote interdisciplinary collaboration and industrial linkage, expand the application of microalgae in emerging fields such as plant-based foods and cellular agriculture, and validate their health benefits through clinical studies to promote microalgae as an integral part of the next generation of sustainable food systems.

## Data Availability

The current editorial contribution was based on the papers published under the Special Issue ‘Microalgae in Food Systems: From Cultivation to Application’.
